# Using a hybrid demand-allocation algorithm to enable distributional analysis of land use change patterns

**DOI:** 10.1371/journal.pone.0240097

**Published:** 2020-10-15

**Authors:** Evan B. Brooks, John W. Coulston, Kurt H. Riitters, David N. Wear

**Affiliations:** 1 Department of Forest Resources and Environmental Conservation, Virginia Tech, Blacksburg, Virginia, United States America; 2 USDA Forest Service, Southern Research Station, Blacksburg, Virginia, United States America; 3 USDA Forest Service, Southern Research Station, Research Triangle Park, North Carolina, United States America; 4 Nonresident Senior Fellow, Resources for the Future, Washington, DC, United States America; Northeastern University (Shenyang China), CHINA

## Abstract

Future land use projections are needed to inform long-term planning and policy. However, most projections require downscaling into spatially explicit projection rasters for ecosystem service analyses. Empirical demand-allocation algorithms input coarse-level transition quotas and convert cells across the raster, based on a modeled probability surface. Such algorithms typically employ contagious and/or random allocation approaches. We present a hybrid seeding approach designed to generate a stochastic collection of spatial realizations for distributional analysis, by 1) randomly selecting a seed cell from a sample of *n* cells, then 2) converting patches of neighboring cells based on transition probability and distance to the seed. We generated a collection of realizations from 2001–2011 for the conterminous USA at 90m resolution based on varying the value of *n*, then computed forest area by fragmentation class and compared the results with observed 2011 forest area by fragmentation class. We found that realizations based on values of *n* ≤ 256 generally covered observed forest fragmentation at regional scales, for approximately 70% of assessed cases. We also demonstrate the potential of the seeding algorithm for distributional analysis by generating 20 trajectories of realizations from 2020–2070 from a single example scenario. Generating a library of such trajectories from across multiple scenarios will enable analysis of projected patterns and downstream ecosystem services, as well as their variation.

## Introduction

Land use choices have modified landscapes across the United States (US) and the world ([1, 2]). As societal needs change, the priorities underlying these choices can shift, yielding a land use change system that is driven by socioeconomic values. There are abundant examples of developed land uses displacing other rural land uses such as forest and agriculture (e.g., [3,4]) and likewise shifting rural land uses (e.g., [5,6]) as different rural land use choices become more or less economically attractive. These land use changes alter the lands’ ability to provide ecosystem services, so projecting land use change informs potential land use policies and ecosystem services [7].

The scale at which land use change can be modeled depends in part upon the scale of the response and explanatory data available. In the US, Alig et al. [8], Wear [9], and Mihiar and Lewis [10] estimated econometric models of land use change at the county scale (counties are political or administrative division of states). This choice of scale arises because key observed or estimated variables such as land use change, population, income, and rural land rents are often only available in a consistent fashion at that county scale. Further variables needed for projections, such as population and income, are also generally available at the county or broader scale.

For example, land use change models and projections developed in support of the US Forest and Rangeland Renewable Resources Planning Act (RPA) of 1974 are developed at the county scale [9]. The RPA requires a periodic assessment of the current and potential future conditions of forest and rangelands of the US, and land use change defines a critical mechanism that influences both economic and ecological characteristics of forests and associated ecosystem services. Fifty-year, county-level land use projections for the US were used in the RPA 2010 assessment [11]. While this scale can appropriately inform the analysis of potential shifts in some ecosystem services (e.g., carbon sequestration), the analysis of other ecosystem services (e.g., water quantity [12]) and ecological indicators (e.g., forest fragmentation [13]; biodiversity [6,14]) requires finer scale information (e.g., 30–90 meter rasters). Our goal is to develop a technique to translate coarse-scale land use change projections to a finer spatial grain in support of the RPA assessment. We focus on applications in the US but expect the results of this research to be applicable globally.

While there are a wide variety of land use change models available as the result of broad interest and research, which can be categorized along economic/non-economic, spatially-explicit/aggregated, and empirical/process-based lines [15,16]; demand-allocation methods [17–21] are particularly well-suited to situations where a projected land use change is available at a coarse scale and the intent is to downscale the projection. Demand-allocation models tend to input exogenously generated transition quantities (or quotas) for a given area (e.g., county), for example via socioeconomic models [10,18]; then allocate those quotas within the study area [22]. This allocation can be driven by empirically estimated transition probability models (e.g., [18,19]), or it can be the result of process-based models such as cellular automata or related hybrids [21,23–25]. Such models can be employed using fuzzy or crisp logic [26].

The RPA county-scale land use projections are well-suited for demand-allocation approaches, but the continental scope of the assessment and fine grain of the spatial output (in this case, 30–90 meters) make process-based models difficult to parameterize consistently over large areas. We therefore focus on empirical approaches, which typically blend contagion-based processes that allocate transitions to a new land use class near areas where that land use class currently exists [27]), with random processes that ignore spatial structures altogether. Seeding algorithms, a subset of demand-allocation algorithms, make use of use both contagious and random processes [18,27,28]. In seeding, the underlying land use change probability surface is randomly sampled, and the cell (pixel of a raster) with the highest conversion probability of the sample is chosen as a ‘seed’ cell. A patch of cells is then grown semi-contagiously from the seed (e.g., [18]), so that patch properties (such as patch sizes for the realized population of patches) correspond to empirical distributions of those properties. Seeding algorithms such as FORE-SCE [28] have been used to model land cover change at national scales, both historically [29] and into the future [19,30].

We recognize the high level of uncertainty in identifying the specific locations of change. Ahmed et al. [31] noted that pixel by pixel comparisons do not necessarily capture the qualitative similarities, such as pattern, between rasters. Aguejdad et al. [17] observed that when the spatial configuration of the landscape is of interest to the end user, the simulation of the landscape pattern is crucial. Additionally, while scenario-based projections make it possible to attribute inter-scenario variation to the differences in the exogenous transition quotas [32], the stochastic nature of seeding algorithms implies a distribution of landscape patterns and metrics based on the varying realizations [18]. Accounting for this within-scenario variability is necessary when assessing the impact of fine-grain landscape projections on ecosystem services. Hence, there is a need to generate not one, but a suite of outputs corresponding to the realizations of both multiple scenarios and multiple possible futures within any given scenario. To our knowledge, no one has accomplished this in support of a large-scale (i.e., national) project. These multiple realizations would support distributional analyses so that uncertainty can be adequately displayed, but they require their parent land use change models to flexibly input multiple similar data streams, yet generate outputs at a fast-enough rate to meet project timelines.

In this study, we introduce and demonstrate an empirical seeding demand-allocation algorithm by making 90m spatial realizations of RPA county-level land use change projections. This seeding algorithm is designed specifically to 1) model a continuum from simple random allocation to contagious allocation, allowing an end user to influence how strictly the conversions should follow the underlying probability surface; and 2) efficiently generate a broad collection of spatial realizations to fuel distribution-based analyses of projected scenarios. The seeding algorithm employs a data-driven process for growing contiguous land use change patches from initial seed cells, which allows for more realistic land use change propagation patterns. Generating a library of multiple realizations yields both projections of fine-scale future land use patterns and a distribution-based metric of confidence (or susceptibility) for given areas to undergo land use change. Although our assessment focus is on forest fragmentation, the interdependency of forest land use with urban, agriculture, and other land uses requires the simultaneous projection of each of these land use classes. We generate fine scale rasters because we are primarily interested in their ability to mimic the aggregate landscape pattern; therefore, the assessment of our algorithm’s performance is by comparing its outputs with observations in terms of forest fragmentation at county, state, and regional scales.

## Methods

### Overall framework

**Fig 1:**
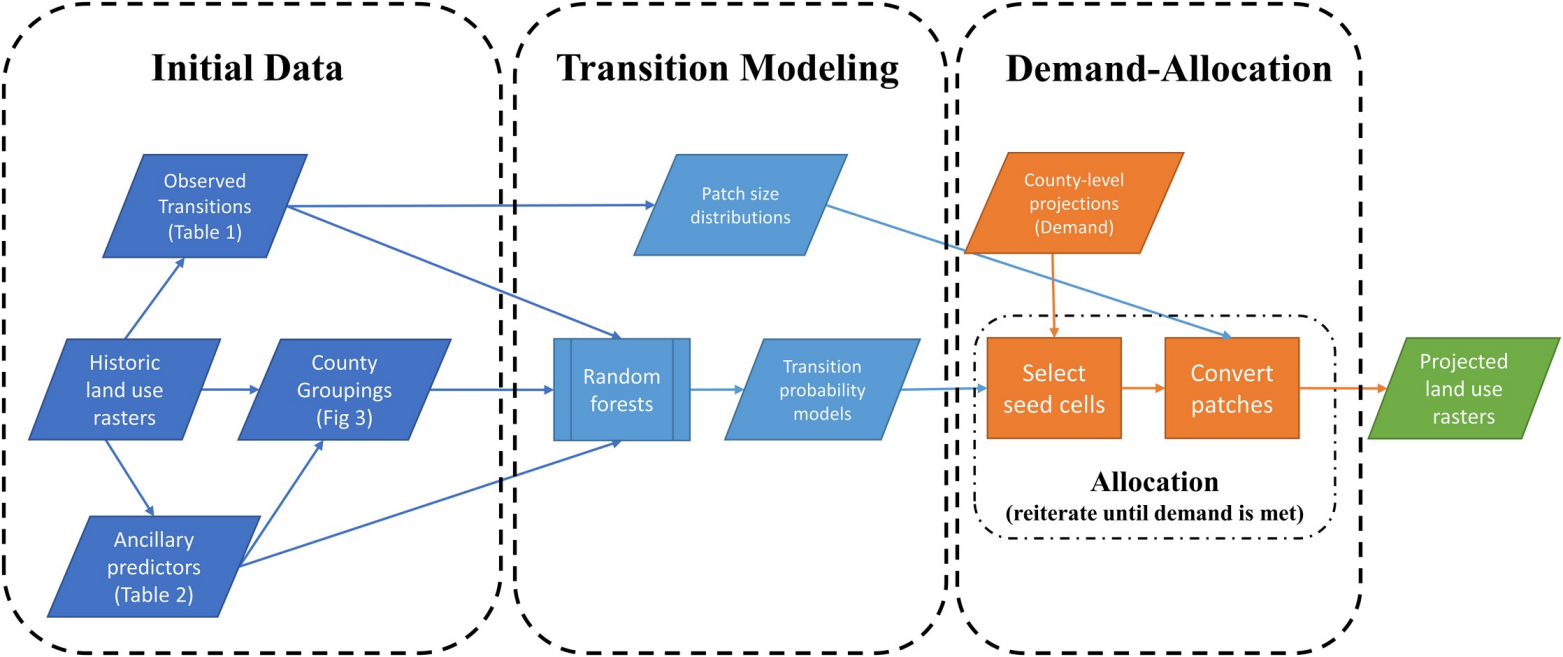
Methodology framework. Key components of the demand-allocation algorithm presented in this study.

In the following methods subsections, we detail our methodology for creating spatial realizations of projected land use from county-level projections (Fig 1). We begin with a description of our reference datasets and other ancillary data used, as well as a description of datasets derived from this initial data. Next, we briefly review the random forests algorithm before describing its use to model land use transition probabilities. Then we describe our specific demand-allocation algorithm, in particular, 1) seed cell selection and 2) patch conversion. Once the algorithm is described, we focus on analyzing the efficacy of the resultant spatial realizations when varying a key tuning parameter, followed by a demonstration of the algorithm through multiple time steps.

**Fig 2:**
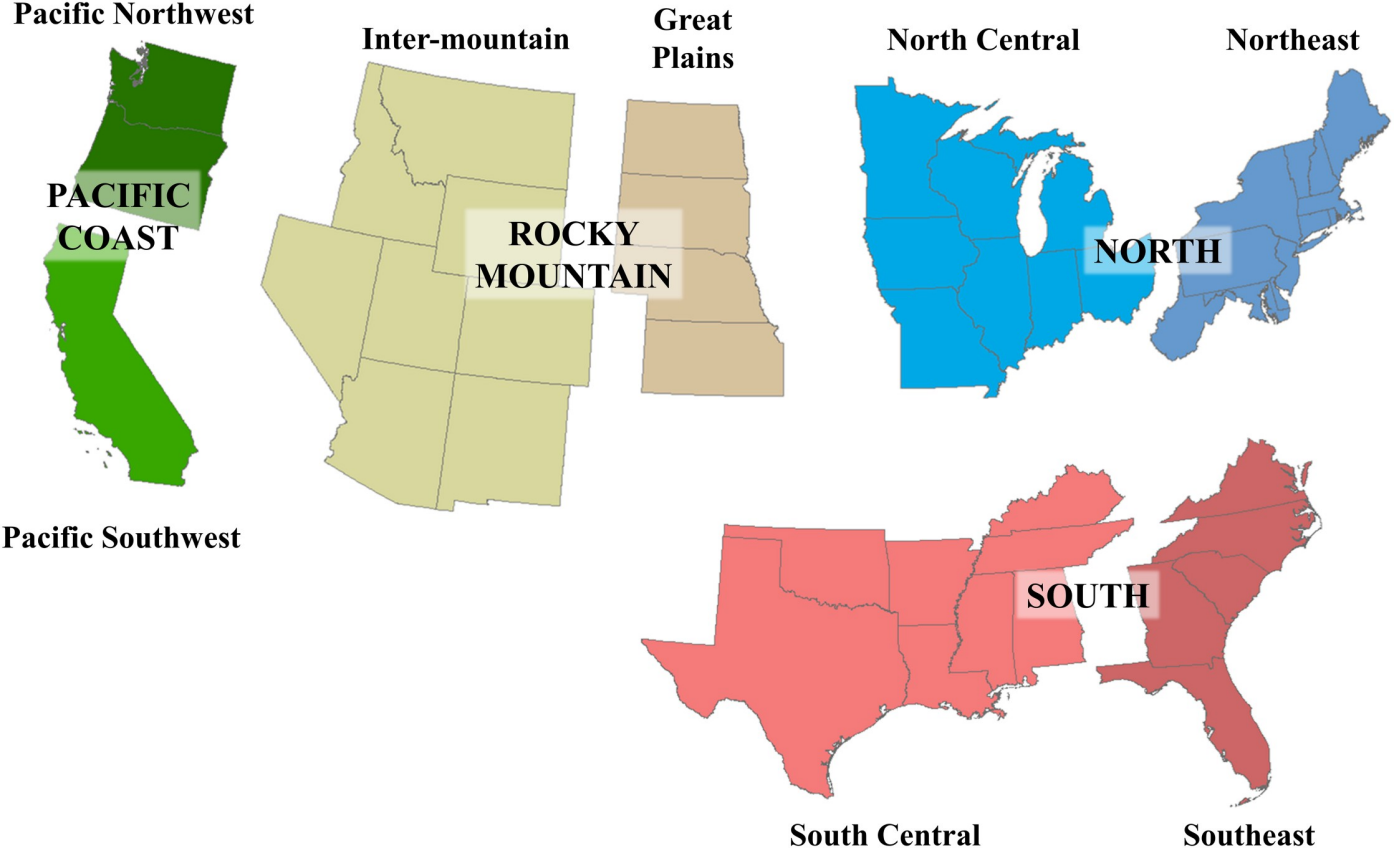
Study area. The conterminous United States (CONUS), as divided into the eight regions used by the Resource Planning Act (RPA) assessments. State boundaries are based on data freely available through the US Census Bureau (https://www.census.gov/geographies/mapping-files/time-series/geo/tiger-line-file.html).

Our study area is the conterminous US (CONUS), represented in this study by a 90m raster with dimensions 51,393 x 32,426 pixels. The raster was partitioned into 3,075 unique county units based on an overlay with US Census data, and grouped into eight regions conforming to RPA conventions (Fig 2). Each county is identified by its Federal Information Processing Standard (FIPS) numeric code. Pixels outside the collective county boundaries were treated as background and excluded from this study.

### Initial data

For our baseline land use data, we adapted the National Land Cover Datasets (NLCD) [33–35] for 2001, 2006, and 2011 into a classification of Developed, Forest, Agriculture, Water, and Other land use classes. To better approximate forested land use from land cover products, we assigned to the Forest class any pixels which were observed to have a forested land cover (i.e., NLCD classes 41, 42, 43, or 90) in any of 2001, 2006, or 2011, while the NLCD land cover in other years was either grassland/herbaceous (NLCD class 71) or shrub/scrub (NLCD class 52). This approach was intended to mimic the successional stages of a replanted forest stand after harvest, which may not have been classified as forest cover in a given year [36]. We treated the resulting 2001 (designated as time 1, or T1) and 2011 (T2) land use rasters as the reference data for all subsequent analysis.

In total, there were 25 possible T1-T2 transitions among the five land use classes. Of these, we only considered a subset (Table 1) based on simplifying assumptions that excluded rare transitions (e.g., Developed to Forest) and transitions prohibited by regulation (e.g., a protected status or federally owned land). Using the reference T1 and T2 rasters, we collated the reference transition patches in tables of total converted area and patch size [37], with one such table per land use transition type (e.g., Agriculture to Developed) per county. We used these tables to both estimate patch size per transition type via a fitted negative binomial distribution, as well as to obtain county-level transition quotas for generating the spatial realizations, to ensure that the projected gross land use areal changes from T1 to T2 would match the observed areal changes. Also, because land use patterns are not the sole driver of land use change decisions, we also assembled a collection of ancillary datasets in raster form, to be used when estimating the transition probability models (Table 2). In addition to land use pattern data derived from the reference T1 raster, these ancillary data included topographic, hydrologic, and legal variables with expected impacts on land use change decisions.

**Table 1:** Transitions between land uses in this study. Only those marked with an X were considered when generating the transition probability model or the spatial realizations.

		**Time 2**				
		**eveloped**	**Forest**	**Agriculture**	**Water**	**Other**
**Time 1**	**Developed**	X				
	**Forest**	X	X	X		X
	**Agriculture**	X	X	X		X
	**Water**				X	
	**Other**	X	X	X		X

**Table 2:** Ancillary datasets used to model transition probabilities.

Variable Type	Scale(s) (90m raster pixels)	Description
**Geophysical**	1x1	Elevation
	3x3, 9x9, 27x27	Maximum slope
	3x3, 9x9, 27x27	Compound topographic index [38]
	1x1	Distance to nearest waterbody
**Legal**	1x1	Protected Areas Database GAP status [39]
**T1 Land use**	3x3, 9x9, 27x27	Percent Developed
	3x3, 9x9, 27x27	Percent Forest
	3x3, 9x9, 27x27	Percent Other
	3x3, 9x9, 27x27	Percent Agriculture
	3x3, 9x9, 27x27	Percent Water
	1x1	T1 land use

**Fig 3:**
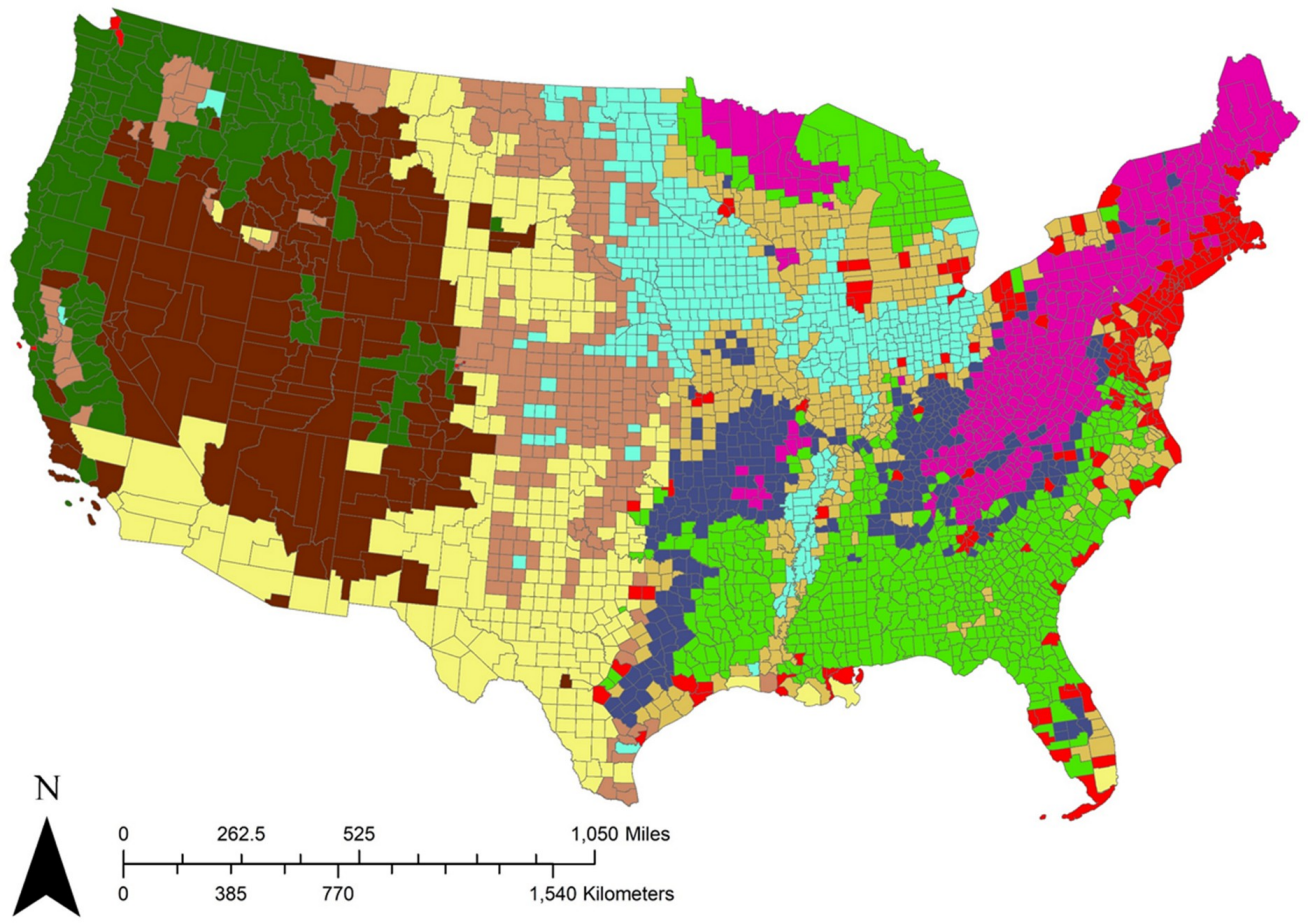
Probability model groupings. The conterminous United States (CONUS) was partitioned into 10 groupings of similar land use patterns for purposes of transition probability modeling. The groupings were derived from a k-means cluster analysis based on per-county land use distribution, elevation and slope, population and income, and geographic location. County feature data is freely available upon request from the USDA Forest Service Forest Inventory and Analysis program (https://data.fs.usda.gov/geodata/edw/datasets.php).

Different areas of the US have historically exhibited different patterns of land use and land use change due to variable geographical, climatic, cultural, and socioeconomic factors. Because those factors do not necessarily follow political boundaries, such as the eight RPA regions of Fig 2, we also partitioned the CONUS into groups of counties (denoted for this study as *groupings*, as opposed to the RPA *subregions* used to aggregate and summarize results) with similar characteristics when estimating land use transition models. The groupings were developed via a k-means cluster analysis that considered similarity of land use distribution, mean elevation and slope (in a 3x3 pixel window), population and income (using 2015 projected data as a proxy, to harmonize with later future projections), and geographic coordinates. We selected a 10-grouping partition (Fig 3), treating each such grouping as a self-contained unit for purposes of modeling transition probabilities. We held the groupings fixed in this study, but for the RPA 2020 assessment, the trajectories of the population and income projections will allow counties which undergo changes (e.g., increased urbanization) to be accordingly reassigned to new groupings.

### Transition modeling

We modeled observed land use class transitions independently for each grouping, using a random forest model. The random forest is an ensemble machine learning approach based on classification and regression tree (CART; [40]) outputs. In general, given a response variable, *y*_*N*×1_, and a corresponding collection of *V* predictor variables, *X*_*N*×*V*_, CART generates a series of recursive partitions, based on splits along the variables in *X* that emphasize within-node purity: that is, each node on the resulting tree is as homogeneous in *y* as possible.

One shortcoming of CART is that it tends to overfit the data, sacrificing model interpretability for explanation of variance in the data. A random forest, an ensemble of *T* CART models (or trees), remedies this through bootstrap aggregating (or bagging). Following the bootstrap notation in Coulston et al. [41], for the *b*^*th*^ tree, let $y_{n\times 1}^{*b}$ be the portion of the original response sampled, where we sample *n* times with replacement from {1, 2, …, *N*}, and let y*^−*b*^ be the portion of *y* not included in the sample. Because we sample with replacement, y*^−*b*^ does not necessarily have *N* − *n* elements. Define $X_{n\times v}^{*b}$ and X*^−*b*^ similarly, by additionally sampling without replacement *v* times from the *V* variables in *X*. Then, construct the corresponding *b*^*th*^ tree via CART and use it to predict the response for the omitted portion, denoted ${\hat{y}}_{\;}^{*-b}$. Repeating this process *B* times results in a random forest where, for any given *y*_*i*_ ∈ *y*_*N*×1_, the predicted value for *y*_*i*_ is the out-of-bag (OOB) mean: ${\hat{y}}_{i}\;=\;\frac{1}{N_{i}}\sum_{\left\{b:y_{i}\in y_{\;}^{*-b}\right\}}^{\;}{\hat{y}}_{\;}^{*-b}$, where $N_{i}\;=\;\sum_{\left\{b:y_{i}\in y_{\;}^{*-b}\right\}}1$ is the number of trees not trained with *y*_*i*_. When *y* is categorical, this mean can be replaced by a majority vote from the trees or, as we did in this study, the proportion of votes for each category can be calculated to obtain the probabilities for selecting those categories. The bagging approach used in random forest also allows for robust error estimation (e.g., OOB error) and measures of variable importance, related to the preponderance of key variables across the forest. We refer the interested reader to [42,43] for more complete details regarding random forests.

In this study, we constructed random forest models to obtain the probabilities of each non-water land use (Agriculture, Developed, Forest, and Other) transitioning into the others, with the exception of Developed, which we assumed not to transition to anything else. Using transition type (Table 1) as the response, we constructed by compiling 500 randomly sampled observations of each transition type within each county grouping; each element of *y* was a categorical gross transition type (i.e., Agriculture to Forest was a distinct transition type from Agriculture to Developed, or Other to Forest). For the explanatory variables, *X*, we used the T1 land use state, as well as a collection of geophysical, T1 land use pattern, and legal variables (Table 2). We then constructed a random forest model and predicted the probabilities associated with each type of land use conversion, for each cell in the grouping: $P\;=\;\{p_{il_{1}l_{2}}\}$, where *i* indexes the cells, *l*_1_ is the given T1 land use class, and *l*_2_ is the target T2 land use class. Taken together across all 10 groupings, these probabilities can be interpreted as a wall-to-wall, multilayered raster with each layer corresponding to the probability of cells undergoing a particular transition. (In practice, we applied the random forest models to each county separately.).

### Demand-allocation

Let $C\;=\;\{c_{1},c_{2},\ldots ,\;c_{N}\}$ denote the *N* non-water cells of a given county, classified into the land uses of L={d,f,a,o} so that C comprises *N*_*d*_ developed, *N*_*f*_ forest, *N*_*a*_ agriculture, and *N*_*o*_ other cells; for example, if the first indexed cell of the county is developed, then *c*_1_ = *d*. Similarly, let $Q\;=\;\{\begin{array}{cccc} q_{dd} & q_{df} & q_{da} & q_{do}\\ q_{fd} & q_{ff} & q_{fa} & q_{fo}\\ q_{ad} & q_{af} & q_{aa} & q_{af}\\ q_{od} & q_{of} & q_{oa} & q_{oo} \end{array}\}$ be the collection of given gross transition quotas per class for the county. For this study, we were only interested in transitions into developed, as well as transitions between the other classes, simplifying Q to $Q\;=\;\{q_{fd},\;q_{ad},\;q_{od},\;q_{af},\;q_{of},\;q_{fa},\;q_{oa},\;q_{fo},\;q_{af}\}$. Note that the county may also be partitioned such that $C\;=\;C_{d}\cup C_{f}\cup C_{a}\cup C_{o}$, where, for example, $C_{d}\;=\;\{c_{d_{1}},c_{d_{2}},\ldots ,\;c_{d_{N_{d}}\;}\}$ represents the collection of developed pixels in the county at time 1. Additionally, let $P\;=\;\{p_{il_{1}l_{2}}\}$ denote the transition probabilities for the *i*^*th*^ cell from that county’s associated random forest model; for example, *p*_1*df*_ is the probability for the first indexed cell to transition from developed into forest at time 2. As $p_{il_{1}l_{2}}\;=\;0$ trivially whenever *l*_1_ ≠ *c*_*i*_, let $\hat{P}\;=\;\{{\hat{p}}_{il}\}$, where ${\hat{p}}_{il}\;=\;\sum_{l_{1}\in L}p_{{il}_{1}l}$. Thus, ${\hat{p}}_{il_{2}}$ represents the generalized probability of converting the *i*^*th*^ cell to class *l* at time 2.

For a target time 2 class *l*, consider the target quotas $\{q_{ml}\}\in Q$, where *m* can be any valid time 1 donor class (e.g., forest, agriculture, or other, provided the donor is not the same as the target class). From $\left\{{\hat{p}}_{il}\right\}$, sample *n* times without replacement with equal probability, and select the seed cell, *i*′, such that ${\hat{p}}_{i^{'}l}$ is the maximum such value in the sample. (If multiple values tie for the maximum, randomly sample a single cell from these.) Then, note the value of *c*_*i*′_, denoting it as *m*′, and reference the negative binomial distribution fitted from the corresponding patch sizes in the reference transition data for the county. For example, if *m*′ = *f* and *l* = *d*, reference the negative binomial distribution fitted from all observed patch sizes from forest to developed land use in the reference dataset. From the appropriate negative binomial distribution, generate a random number, *k*, to be the patch size surrounding the seed cell, *i*′. In practice, we require the patch size to be an odd number of cells for computational simplicity; if *k* is even, adjust its value upward by adding 1. Also note that in the event there was no negative binomial distribution fitted for the transition in question (i.e., no such transitions were observed in the reference data), we set *k* = 1 and proceeded.

Denote the patch window, K, as the collection of cells within the county such that their annular distance to the seed cell is less than or equal to the radius, $r\;=\;\frac{k-1}{2}$, where annular distance between cell *j* and cell *i*′ is defined as *dist*(*i*′,*j*) = max(abs(*x*_*j*_ − *x*_*i*′_),abs(*y*_*j*_ − *y*_*i*′_)), where *x* and *y* represent the northing and easting of the cell locations within the county. For ease of notation and without loss of generality, we can reindex the cells in the patch window more geographically, as

$$K_{k\times k}\;=\;[\begin{array}{ccccc} c_{(-r)r} & \cdots & c_{0r} & \cdots & c_{rr}\\ \vdots & \ddots & \vdots & ⋰ & \vdots \\ c_{(-r)0} & \cdots & c_{i^{'}}\;=\;c_{00} & \cdots & c_{r0}\\ \vdots & ⋰ & \vdots & \ddots & \vdots \\ c_{(-r)-r} & \cdots & c_{0(-r)} & \cdots & c_{r(-r)} \end{array}],$$ (1)

so that *dist*((0,0),(*i*,*j*)) = max(abs(*i*),abs(*j*)).

**Fig 4:**
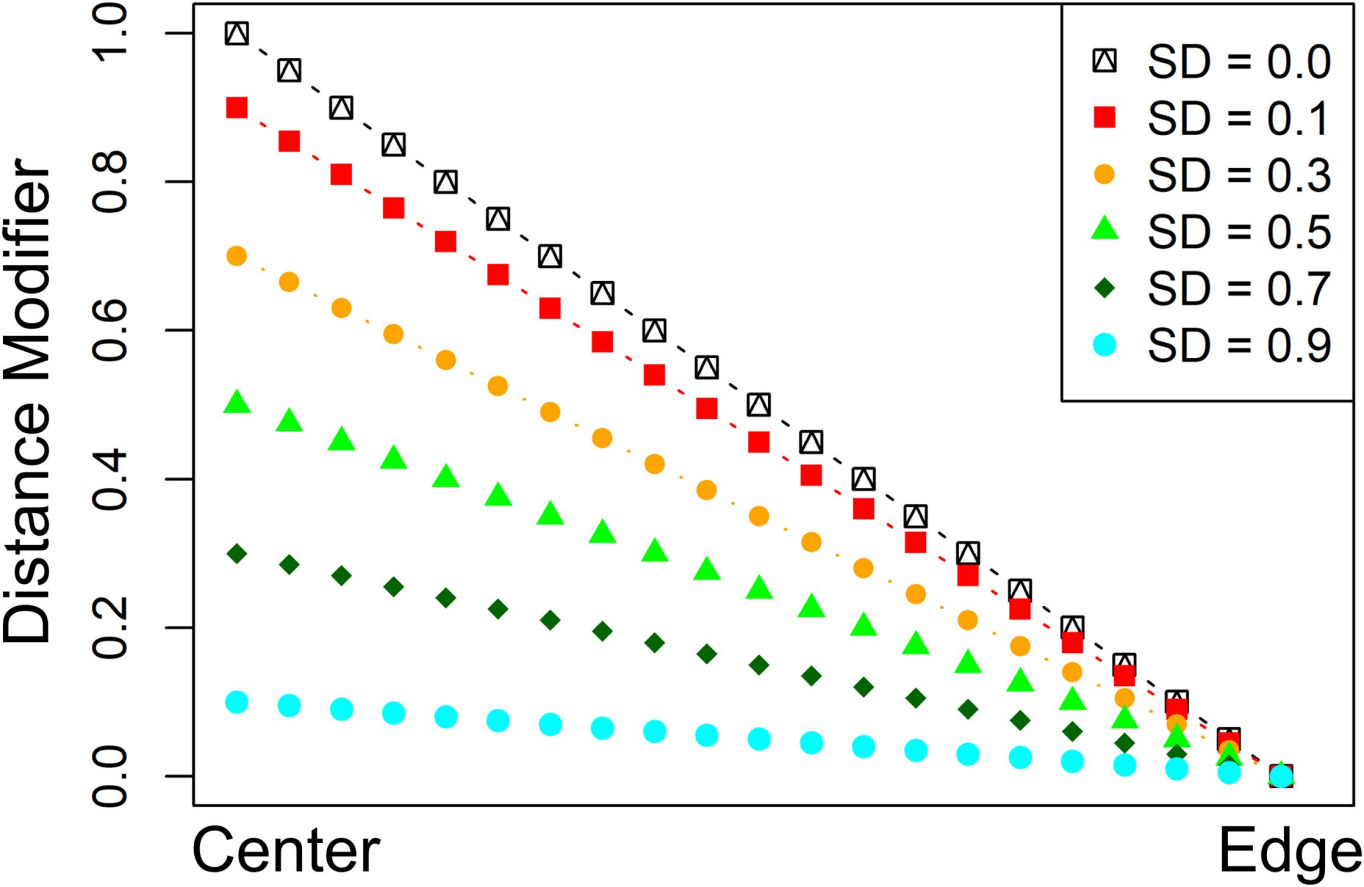
Distance modifier. Distance modifier to pixel transition probabilities, as a function of distance to center and local standard deviation of transition probabilities. Note how as the standard deviation increases, the impact of the distance modifier is lessened.

Then, for all viable cells in the patch window that might transition to class *l* at time 2, that is, all cells $\hat{K}\;=\;c_{ij}\in K$ such that $c_{ij}\in L\backslash \{l,\;d\}$ (since *p*_idl_ = 0 for all *i* and *l*), consider their generalized probabilities of converting to class *l*, reindexing for convenience as ${\hat{P}}_{\hat{K}}\;=\;\{p_{ij}\}$, where $p_{ij}\;=\;{\hat{p}}_{*l}$ for the *^th^ cell corresponding to $c_{ij}\in \hat{K}$. That is, each cell within the patch window that is not currently in the target class, *l*, and has a nonzero probability of converting to class *l*, has both an associated probability of converting to class *l* and an annular distance to the seed cell at the center of the patch window. From these candidate cells, $\hat{K}$, we select *k* cells to convert to class *l*. To ensure that we concentrate converted cells near the seed cell (and thus avoid converting only cells not adjacent to the seed cell), we modify the values of ${\hat{P}}_{\hat{K}}$ according to the corresponding cells’ annular distance to the seed cell:

$${\hat{P}}_{\hat{K}}^{'}\;=\;\{p_{ij}^{'}\},\;where\;p_{ij}^{'}\;=\;p_{ij}+(1\;-\frac{dist_{ij}}{r})(1-\sigma ),$$ (2)

where *σ* is the standard deviation of the generalized transition probabilities in ${\hat{P}}_{\hat{K}}$. This modification raises the generalized transition probabilities for cells geographically near the seed pixel, relative to the window patch’s edge (Fig 4). When the underlying probabilities are more constant (e.g., all else is equal within the patch window), the distance modifier dominates and cells nearest the seed cell are favored for conversion. Where the probabilities are highly variable (e.g., there are features in the patch window which drive the viability of candidate cells for conversion), the original probability surface retains a stronger influence on the resulting converted patch.

Finally, given ${\hat{P}}_{\hat{K}}^{'}$, we select a number of cells corresponding to $k^{'}\;=\;\text{min}\left(k,\;∥{\hat{P}}_{\hat{K}}^{'}∥,\;q_{*l}\right)$, chosen in descending order of their corresponding probabilities, and convert these cells to class *l*: $C_{K^{'}}\;=\;l$, where $K^{'}$ is the first *k*′ elements of $\hat{K}$ according to descending order of ${\hat{P}}_{\hat{K}}^{'}$. These *k*′ cells are then removed from consideration of further conversion by setting $P_{C_{K^{'}}}\;=\;0$, the corresponding quotas, q_*l_, are updated, and the seeding process begins anew within the target time 2 class, *l*. Once the conversion quota is achieved, we iterate through the remaining target time 2 classes until all quotas for the county are achieved; we then iterate through all counties in the CONUS.

### Assessing choice of seeding sample size

The choice of seeding sample size influences the allocation of converted cells and, consequently, the location of the resulting T2 predicted land use. Because seeding with *n* = 1 is similar to random allocation (aside from growing patches from the seeds) and seeding with *n* equal to the population size is similar to contagious allocation, we expected that forest fragmentation metrics from a range of seeding realizations would encompass the observed forest fragmentation. However, preliminary testing revealed that values of *n* greater than 200 produced highly similar outcomes. Therefore, we generated six CONUS-wide realizations of T2 land use rasters for each case of *n* = 2, 4, 8, 16, 32, 64, 128, and 256, for a total of 6 x 8 = 48 realizations. We compared forest fragmentation for these realizations with the observed T2 land use raster. We used the same underlying transition probability models, based on the county groupings, for all realizations.

**Fig 5:**
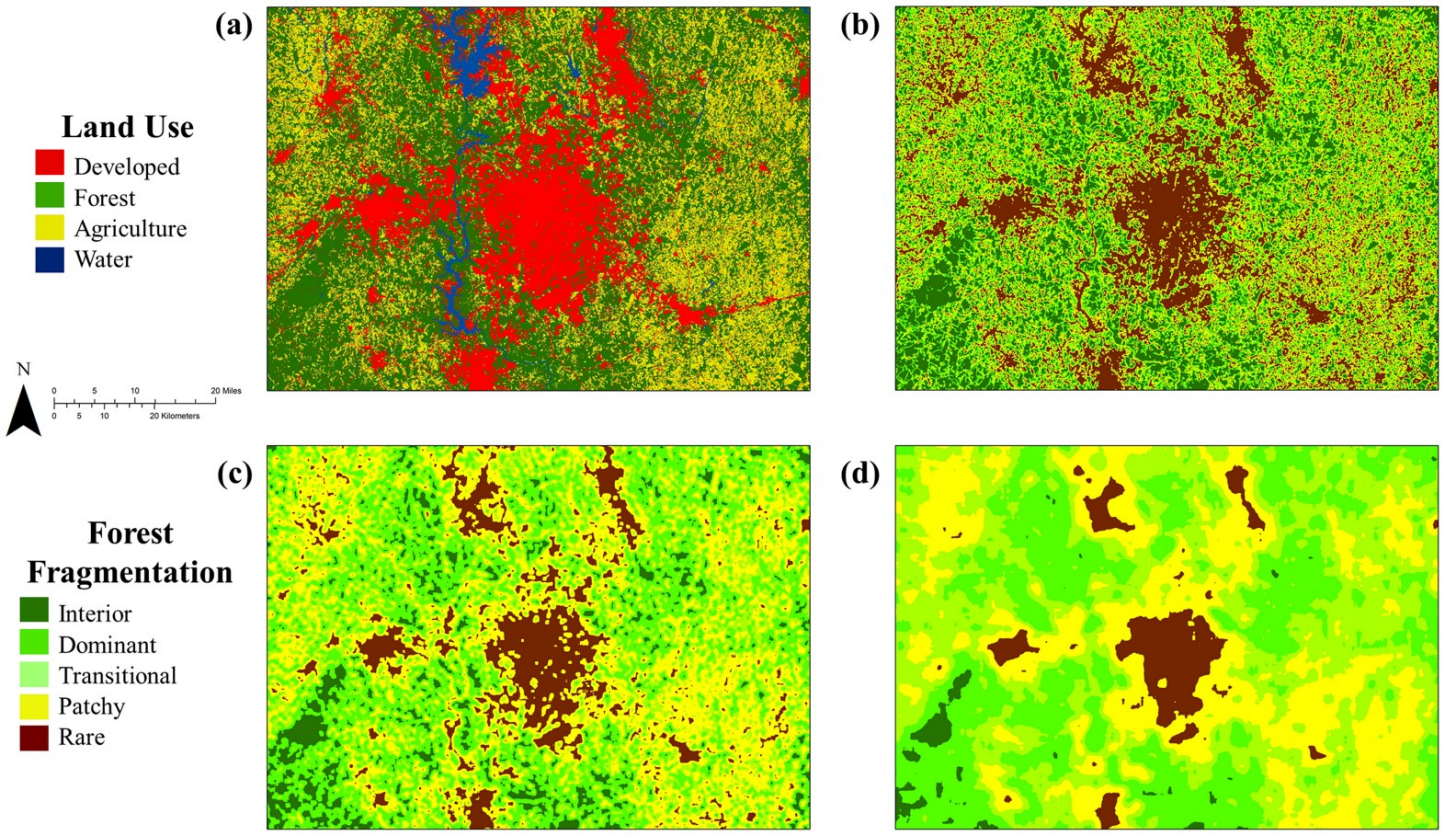
Forest fragmentation classes. (a) Land use around Charlotte, North Carolina, United States, and corresponding forest fragmentation classes for (b) 3x3, (c) 9x9, and (d) 27x27 pixel windows. See Table 3 for definitions for the specific classes.

Irrespective of the method used to generate a raster, forest fragmentation depends on the spatial scale at which it is measured. To measure fragmentation at multiple spatial scales, we computed on each realized raster the percent Forest within windows of 3x3, 9x9, and 27x27 90m cells (7.3, 65.6, and 590.5 hectares, respectively) and assigned the result to the cell at the center of the window. Using a classification framework (Table 3, Fig 5) similar to that of [44], we then compared areas of each forest fragmentation class with respect to the reference T2 fragmentation, against the corresponding areas from the realizations. In each case, we checked whether the observed forest area within each class was covered by the distribution of areas from the various realizations. Because we were interested in focal-class forest fragmentation and not landscape-scale fragmentation, we only considered forest fragmentation metrics for pixels classified as forest at each date. For example, a developed pixel entirely surrounded by forests was not considered, but a forested pixel entirely surrounded by developments was. While results were produced at the county level, for simplicity we aggregated results according to the eight RPA subregions (Fig 2).

**Table 3:** Forest fragmentation classes used, based on percent forest within various window sizes.

Fragmentation Class	Percent Forest
**Interior (including intact)**	90–100%
**Dominant**	60–89%
**Transitional**	40–59%
**Patchy**	10–39%
**Rare (including none)**	0–9%

### Demonstrating multiple projected time steps

As an additional demonstration, we generated projected land use rasters in decadal time steps. These test realizations were based on a land use raster representing a 2020 starting point; the base raster was constructed from NLCD data from 2001–2016, as well as data developed by the US Department of Agriculture Forest Service in support of the RPA 2020 assessment [45]. County-level quotas for the land use transitions were independently provided for 2030–2070, covering each county across the CONUS, using econometric models similar to those of Mihiar and Lewis [10]. These models treat land use decisions as a function of comparative rent values for each available land use. The rent values are dependent on socioeconomic factors as well as climate factors, so that as these factors change, the proportion of land in a given county dedicated to each use will change accordingly. For the current demonstration, we used a baseline model that held climate inputs fixed and employed a single socioeconomic storyline, as downscaled by Wear and Prestemon [46]. We generated 20 spatial realizations according to these quotas via the process described in the preceding subsections, varying the value of the seeding sample size, *n*, between 20 and 200, then computed forest fragmentation classes and areas using the same methods as the previous subsection. We summarized the results by RPA region as before, then produced trajectories through time for each forest fragmentation class and RPA region.

## Results

### Transition modeling

**Fig 6:**
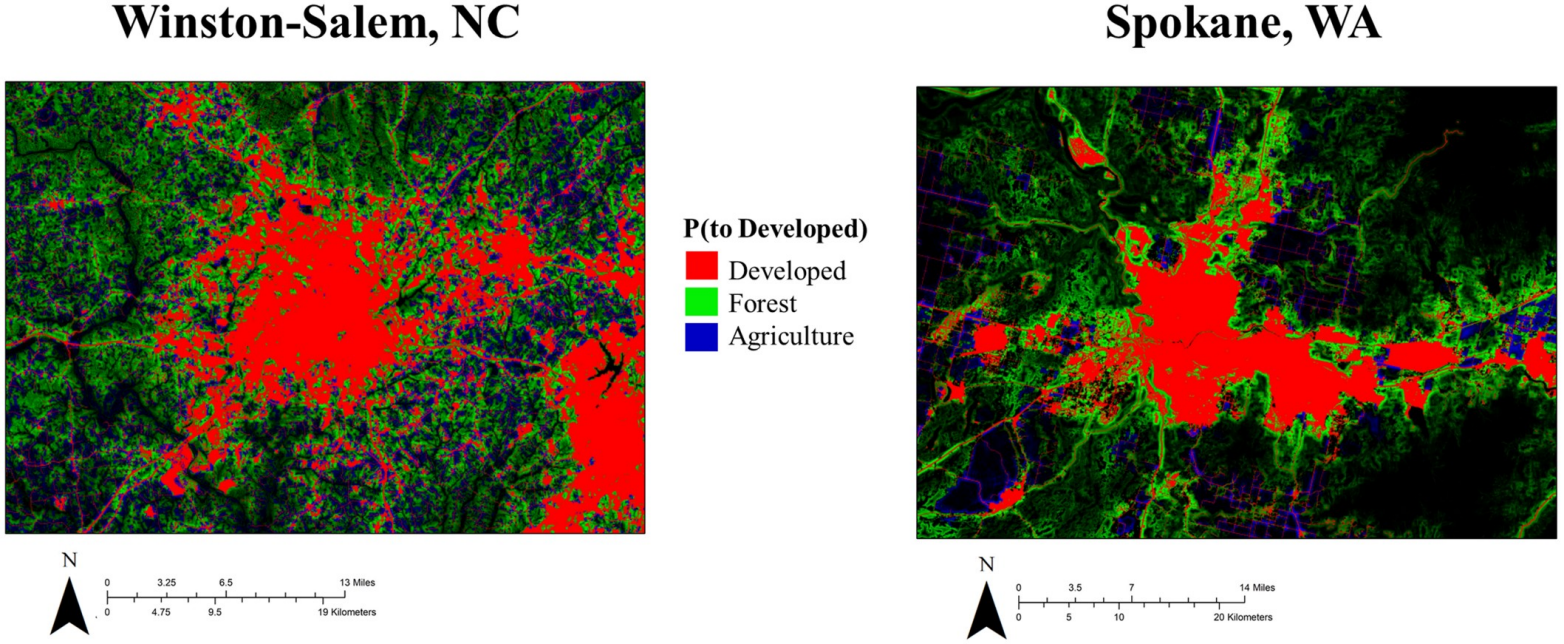
Transition probability rasters. Example probability rasters of transitions to the Developed land use class from Developed (red), Forest (green), and Agriculture (blue) land use classes. The probability of transitioning from Developed to Developed is set at 1, so the red pixels are intended here as a convenient reference to previously existing Developed area.

We generated a probability model for each of the county groupings using the random forest approach. OOB classification error varied by grouping, ranging from 25.9% to 41.9%. Explanatory variable importance was generally similar for all groupings, with T1 land use (1x1 pixels), percent land use in 3x3 windows, and elevation consistently ranking among the four most important variables for each model. Fig 6 shows rasterized results generated from such probability models, where each color corresponds to the probability of going from a specific land use class to Developed; in this case, the relationship of transition probability to the predictor of local percent Developed is demonstrated.

### Assessing choice of seeding sample size

**Fig 7:**
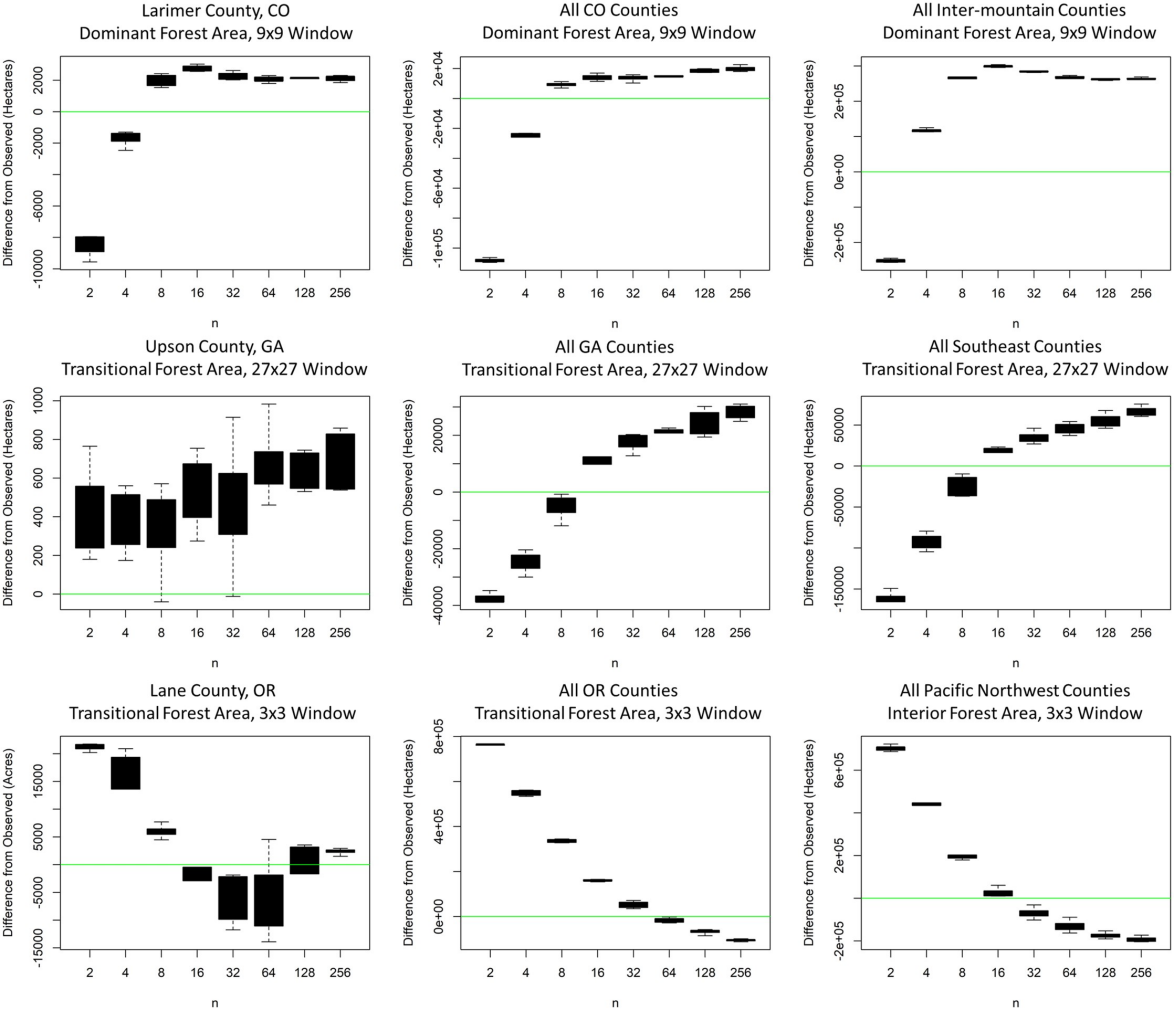
Coverage of observed forest patterns. Differences between the total observed forest fragmentation and that derived from realizations, with respect to choice of the seeding size, *n*, and the scale of aggregation for results. Three example forest fragmentation classes (Table 3; Fig 5) are shown here. The green line represents a difference of 0 acres between the observed forest area and realized forest area, for forested pixels of the given fragmentation class at the given scale. Each boxplot represents six realizations per choice of *n*.

We compared the T2 forest fragmentation from the seeding realizations to the observed T2 forest fragmentation. In most cases, we found clear trends relating forest fragmentation classes to the seeding sample size, *n*, at multiple scales of aggregation (Fig 7): modeled values started near one percentage, then shifted toward a different percentage with a relatively stable variance between choices of *n*. Generally, larger values of *n* in this range produced similar results, a finding consistent with our preliminary tests which yielded little variation in classified forest area between realizations using *n* larger than 200.

**Fig 8:**
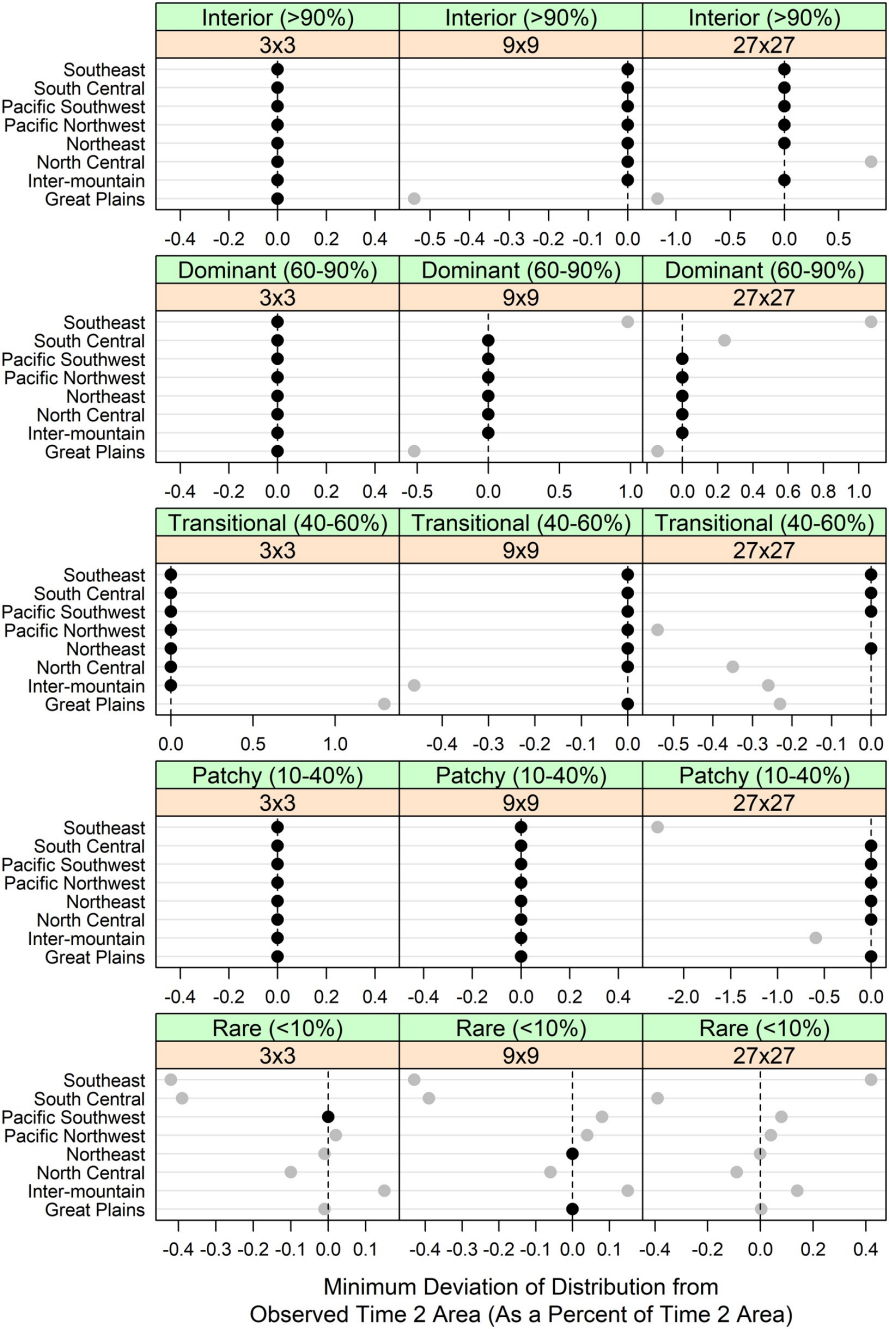
Coverage of forest patterns by RPA region. Coverage of observed forest areas by fragmentation class, by window size (when determining percent forest) and RPA region. Values shown represent the minimum deviation of the forest area from the 48 realizations, as compared to the observed time 2 forest area. Values of 0 indicate that the observed time 2 forest area was contained within the distribution of the realized forest areas.

Aggregating across the six realizations from each of eight choices of *n*, the distribution of seeding-derived fragmentation (48 realizations total) covered the observed time 2 fragmentation at the RPA subregional level and fragmentation window size for 83 of the 120 cases (~70%; Fig 8). Excluding the rare forest cases, coverage was ~83%. In cases where the 48 realizations did not cover the observed time 2 fragmentation for a given window size and subregion, the minimum deviation between observed and one of the realizations was typically less than 0.5% of the observed time 2 area. Thus, generating a distribution of realizations, with variable adherence to contagious seeding, enabled coverage of the observed forest fragmentation with good confidence.

### Demonstrating multiple projected time steps

**Fig 9:**
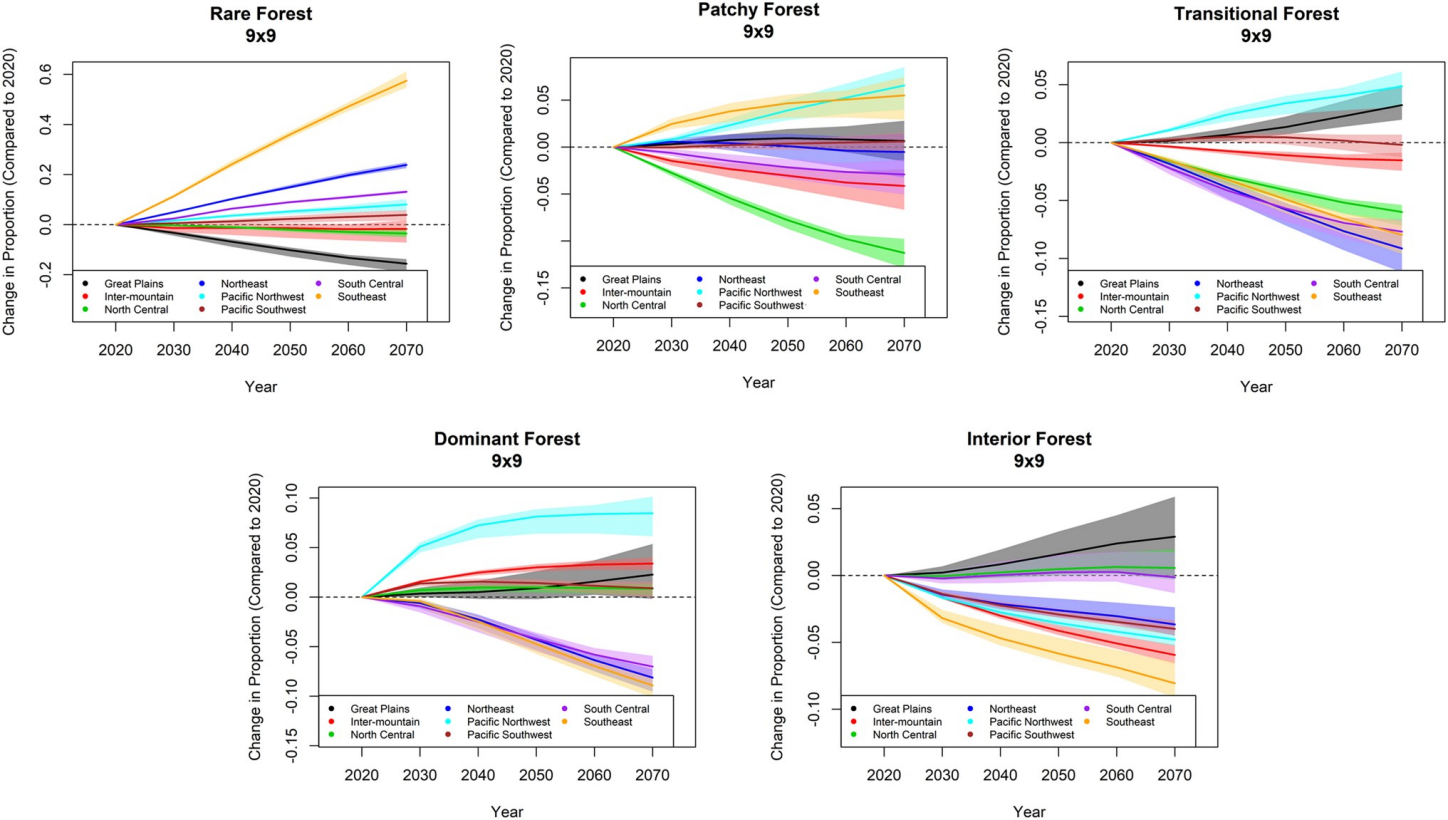
Forest pattern trajectories. Projected change in forested area from 2020–2070, by forest fragmentation class (Table 3; Fig 5) and RPA subregion (Fig 2). Change is given as a percentage of the 2020 area. The lines represent the median value across 20 realizations; the polygons represent the corresponding inter-quartile ranges.

We also demonstrated the utility of having multiple realizations over time, drawing from a single scenario. This approach enables comparison of regional effects, with realization-based uncertainty included. For example, Fig 9 shows trajectories of percent change (relative to the 2020 baseline) of the five forest fragmentation classes, across each of the eight RPA subregions. Inter-realization variation is given in the shaded polygons, representing the first and third quartiles of values from across the 20 realizations used. A rich variety of regional differences and patterns is immediately evident; for example, the amount of dominant forest (60–90% forest in the forested pixel’s neighborhood) in the Pacific Northwest is projected to increase between 6–10% by 2070 under this scenario, with the majority of that increase occurring by 2040. This relative increase is notably greater than that of any other RPA subregion, and based on the other panels in Fig 9, it is clear this increase is coming from a corresponding decrease in interior forest (90–100% forest in the forested pixel’s neighborhood). As another example, in the Southeast the increase to rare forest (a proxy indicator of increased urbanization in that heavily forested subregion) is approximately 60% with low variation, implying with the other panels that (in this scenario) the Southeast is expected to undergo deforestation (loss of dominant and interior forest on the order of 5–10% each) to fuel this transition.

## Discussion

Our goal was to develop and assess a hybrid demand-allocation algorithm for translating county-level land use projections into fine-grain realizations. We demonstrated our approach by evaluating projections of forest fragmentation derived from gross land use projections. We acknowledge the uncertainty in predicting future locations of land use change [17,31] and hence employ a multiple-realization approach where the distribution of potential future conditions can be assessed. Because our focus was not on pixel-specific accuracy, we note that our algorithm’s realizations may best be used when pixel-based pattern metrics are aggregated to county- or coarser-level summaries, as in our results (Figs 7–9).

Because of the obvious links between land use decisions and climate change [47–49], land use projection efforts such as the RPA 2020 assessment are intextricably tied to climate change projections [11,19,50–52]. These projection studies are most typically based on the Intergovernmental Panel on Climate Change (IPCC) scnearios, which combine radiative forcing and socioeconomic trajectories [53]. Scenario-based projections are desirable because the range of futures is simultaenously simplified yet vetted, allowing the end user to focus on the impacts of the various scenarios in their field of interest without devising and defending those scenarios from scratch. Similarly, scenario-based analyses allow separation of variability between the scenario and the analysis [32]. Additionally, the widespread use of the IPCC scnearios permits a common reference framework, by which disparate studies may be compared and synthesized [53]. In this study we used empirical observations to inform our land use transitions, but we also demonstrated the potential of our algorithm to generate within-scenario and between-scenario variability through projected trajectories.

The most important distinction of our seeding algorithm from other contemporary seeding algorithms (e.g., [18,19]) is that we specifically designed it to generate multiple realizations, via a tunable stochastic process, to create a library of projected allocations, as opposed to a single or small number of outcomes (e.g., one per scenario). This distributional capability has immense utility. When projecting across multiple time steps, we are interested not only in the spatial patterns of land use change but the timing and variability of those patterns. What locations are most susceptible to change? Are there multiple storyline paths to similar future patterns? What is the likelihood of significant or substantial change in fragmentation over time? A distributional approach addresses these questions by generating multiple, equally plausible realizations as a foundation for hypothesis testing. Accordingly, our seeding algorithm makes simplifying assumptions (e.g., a basic probability model generated from a short list of common, spatially explicit drivers of change, such as elevation and slope) in exchange for the greater computational efficiency to produce libraries of rasters at the finer scales (e.g., 30–90 meters) needed for many ecological service and indicator analyses (e.g., [6,12–14,54]).

For example, in support of the RPA 2020 assessment, our algorithm is used to generate spatial realizations from county-level land use projections made by methods similar to Mihiar and Lewis [10]. The RPA 2020 assessment is focused on 20 curated scenarios [55], each one representing a particular combination of climate influence [56] and downscaled socioeconomic storylines from the IPCC [46]. Each realization represents a five-raster trajectory (2030–2070, in decadal steps) from a 2020 CONUS-wide baseline raster at 90m spatial resolution. By varying the seeding sample size randomly from 20 to 200, our algorithm produces a collection of 20 realizations (100 rasters) per scenario, analogous to the data generated to support Fig 9. With available computing resources, it takes our algorithm approximately three days to produce one such collection. The end result will be 420 realizations (20 realizations per 20 scenarios, plus one comparative scenario holding climate effects fixed), enabling distribution-based analyses of landscape pattern projections with the capacity to compare climate-, socioeconomic-, and allocation-based effects on ecosystem indicators and services.

One aspect of this distribution-oriented approach is controlling for the variation of the resulting spatial realizations. Aquilué et al. [18] assessed the interaction between the tuning parameters in their seeding algorithm; these parameters were more focused on patch growth after the initial seeding. The primary tuning parameter in our seeding algorithm is *n*, the seeding sample size, which impacts the spatial distribution of seed cells. The choice of *n* can be conceptualized as placing the realizations on a continuum between contagious and random allocation. If *n* = 1, then seed selection is randomly allocated; if *n* is the number of cells in the study area, then seed selection is contagious. While this conceptualization has limits (in our seeding algorithm, contiguous pathces are grown around the seed cells), one may intuit that larger values of *n* tend to produce more contagiously-allocated realizations than small values of *n*. In practice, this effect is constrained to lower values of *n*: we found that values of *n* less than 256 were generally sufficient to obtain the range of parameter-based variation (Fig 7), and preliminary tests with *n* greater than 256 suggested negligible additional variation with respect to projected forest fragmentation. However, the impact of varying *n* produced more variation in the resulting forest fragmetnation projections than did replicates using the same value (Fig 7). Thus, in generating multiple realizations within a scenario, we can also let those realizations vary according to allocation pattern, further increasing our ability to capture observed variation (Figs 7–9).

In this study, of the 120 combinations of forest fragmentation, window size, and RPA subregion, the distribution of forest areas the 48 spatial realizations captured the observed time 2 value approximately 70% of the time (Fig 8). However, of the combinations where the observed value lay outside the distribution, over half were for the rare forest class, seemingly independent of the window size used when defining that fragmentation class (Table 3). We speculate that this may have had to do with the algorithm and how it allocated patch size: we modeled patch size with a negative binomial distribution, but to cover the cases where no transitions were observed in a county, we inserted a single pseudo-transition of patch size 1 into each transition table. This would have skewed the resulting patch size distirbutions to smaller values, especially in cases where the patch sizes were generally larger, which might explain the general tendency for our distributions of rare forest areas to miss the observed areas. From a different perspective, of the 37 cases in which the distribution of realized forest areas did not cover the observed area, 8 cases (21%) were from the Great Plains subregion, a considerably higher proportion than the 12.5% one might expect from an even distribution of misses across the subregions. This higher proportion may be accounted for by the relatively nonforested nature of the Great Plains.

Numerous avenues of future research stem from our results, many of which focus on adjustments to the algorithm. For example, all realizations in this study were based on the same transition probability models (one per grouping (Fig 3), from observed 2001 to 2011 transitions), but the stability of such empirically-based transition models over time is unknown at best [19]. With the recent release of the NLCD 2016 dataset [57], it is now possible to prepare additional land use data and compare models estimated from the decadal time steps of 2001–2011 (as used in this study) and 2006–2016, to get a better sense of transition model stability. As another example, with regard to patch size selection, we have replaced our negative binomial distributions (originally modeled from empirically observed transition patch sizes) by directly sampling from the observed patch sizes. This bootstrapping approach has reduced the number of small patches and is expected to improve the allocation of small-area land use transitions. A third area of future research could be exploring data-driven ways to select the value of *n*; for example, by overlaying empirically observed transitions against the transition modeled probability surface, then estimating which value of *n* is most likely to produce the observed raster. Such a procedure could be done independently by county, and it could possibly be used as an explanatory variable when determining initial groupings to estimate transition probability models. Finally, we used the random forests approach to estimate our transition probability models because random forests can easily and robustly combine disparate data types into a common predictive framework for land use transitions [21]. In this study, we used one transition probability model per county grouping (Fig 3), effectively limiting the variation in projected landscape pattern due to choice of the underlying probability model. In support of a broader, distribution-focused approach, we could explore the use of multiple random forest models estimated from different training datasets, through an approach similar to [41] to add a transition probability model-specific variation component to the scenario- and allocation-based variation.

## Conclusion

Typically, econometric and other land use change models provide projections at relatively coarse scales [9,10,58]. In this study, we presented a stochastic seeding approach to allocating land use transitions at fine-grain scales based on coarser-scale land use projections. This approach was tunable, allowing exploration along the contagious-random allocation continuum. The seeding approach produced time 2 rasters with similar forest fragmentation to that in the observed time 2 rasters. We found that lower rates of seed sampling (*n* ≤ 256) produced a collection of rasters that typically captured the observed fragmentation across multiple fragmentation scales. Such findings were consistent across the conterminous US, suggesting that our seeding method is well-suited for allocation of projected county-level land use transitions at national scales. We used relatively simplified models and coarse land use classes to keep the overall process computationally tractable, because our seeding algorithm was designed to generate libraries of spatial realizations and temporal trajectories to support sensitivity (e.g., vulnerability to/stability against land use change) and other distribution-based analyses.
